# Return to Work Coordination Programmes for Work Disability: A Meta-Analysis of Randomised Controlled Trials

**DOI:** 10.1371/journal.pone.0049760

**Published:** 2012-11-19

**Authors:** Stefan Schandelmaier, Shanil Ebrahim, Susan C. A. Burkhardt, Wout E. L. de Boer, Thomas Zumbrunn, Gordon H. Guyatt, Jason W. Busse, Regina Kunz

**Affiliations:** 1 Academy of Swiss Insurance Medicine, University Hospital Basel, Basel, Switzerland; 2 Department of Clinical Epidemiology and Biostatistics, McMaster University, Hamilton, Ontario, Canada; 3 Clinical Trial Unit, University Hospital Basel, Basel, Switzerland; 4 Department of Anesthesia, McMaster University, Hamilton, Ontario, Canada; University of Toronto, Canada

## Abstract

**Background:**

The dramatic rise in chronically ill patients on permanent disability benefits threatens the sustainability of social security in high-income countries. Social insurance organizations have started to invest in promising, but costly return to work (RTW) coordination programmes. The benefit, however, remains uncertain. We conducted a systematic review to determine the long-term effectiveness of RTW coordination compared to usual practice in patients at risk for long-term disability.

**Methods and Findings:**

Eligible trials enrolled employees on work absence for at least 4 weeks and randomly assigned them to RTW coordination or to usual practice. We searched 5 databases (to April 2, 2012). Two investigators performed standardised eligibility assessment, study appraisal and data extraction independently and in duplicate. The GRADE framework guided our assessment of confidence in the meta-analytic estimates. We identified 9 trials from 7 countries, 8 focusing on musculoskeletal, and 1 on mental complaints. Most trials followed participants for 12 months or less. No trial assessed permanent disability. Moderate quality evidence suggests a benefit of RTW coordination on proportion at work at end of follow-up (risk ratio = 1.08, 95% CI = 1.03 to 1.13; absolute effect = 5 in 100 additional individuals returning to work, 95% CI = 2 to 8), overall function (mean difference [MD] on a 0 to 100 scale = 5.2, 95% CI = 2.4 to 8.0; minimal important difference [MID] = 10), physical function (MD = 5.3, 95% CI = 1.4 to 9.1; MID = 8.4), mental function (MD = 3.1, 95% CI = 0.7 to 5.6; MID = 7.3) and pain (MD = 6.1, 95% CI = 3.1 to 9.2; MID = 10).

**Conclusions:**

Moderate quality evidence suggests that RTW coordination results in small relative, but likely important absolute benefits in the likelihood of disabled or sick-listed patients returning to work, and associated small improvements in function and pain. Future research should explore whether the limited effects persist, and whether the programmes are cost effective in the long term.

## Introduction

Long-term sickness absence secondary to illness or injury is associated with reduced quality of life [1], [2], and considerable socioeconomic costs [3]–[9]. Both patients who are unable to work and the society benefit from return to work (RTW) [2]. However, RTW often requires overcoming challenges, including coping with on-going health problems, re-establishing work functioning, and finding suitable alternative work if a previous job is no longer available [10]. Lack of cooperation between patients, employers, healthcare providers, and insurers may also complicate RTW [1], [10]. The Organisation for Economic Co-operation and Development (OECD) postulated in 2010 that “more people with disability could work if they were helped with the right supports at the right time” through better “cross-agency co-operation” and “systematic and tailored engagement with clients” [1].

Following this intuitively appealing approach, social and private insurers have increasingly implemented RTW coordination services for people receiving wage replacement benefits [11]. RTW coordination, however, demands considerable effort from the affected individual, health professionals, and employers, often without compensation, and is associated with substantial direct costs for insurers. Involved parties thus require reliable information about the effectiveness of RTW coordination to gauge whether RTW coordination is warranted [1].

Existing systematic reviews of RTW interventions have not focused on RTW coordination [12]–[22]. Therefore, we conducted a systematic review and meta-analysis of randomised controlled trials (RCTs) addressing the effectiveness of RTW coordination compared to usual practice on disability, RTW, function, quality of life and satisfaction in employees receiving wage replacements benefits.

## Methods

Document S1 shows the protocol of the review.

### Eligibility Criteria

Eligible studies met the following criteria: (1) random allocation of adult participants to RTW coordination or usual care, (2) inclusion of participants of whom at least 80% were continuously off work (full or part time sick leave or on disability benefit) for at least four weeks and employed at the time of sick listing, and (3) report of disability status or RTW as an outcome. We defined RTW coordination as involving a direct assessment leading to an individually tailored RTW plan implemented by a RTW-coordinator or team who coordinates services and communication among involved stakeholders.

We excluded employer initiated RTW coordination programmes because they typically focus on prevention of sick leave, and encounter fewer barriers in implementing workplace-directed interventions than insurance or third party RTW coordinators.

### Identification of Studies and Data Collection

We carried out a systematic search of MEDLINE, EMBASE, CINAHL, PsycINFO, and the Cochrane Central Register of Controlled Trials from inception to April 2, 2012. Our search strategy combined possible synonyms of RTW coordination (e.g. case management or multidisciplinary rehabilitation), sick leave and disability with a filter for RCTs (see Document S2). We screened reference lists of relevant articles to identify additional eligible trials. Two reviewers independently and in duplicate screened titles and abstracts in any language, reviewed articles in full text, and extracted data from eligible trials. They resolved discrepancies by discussion to achieve consensus. We contacted study authors if information about eligibility criteria, methodological components, or outcome data was incomplete or conflicting.

### Assessment of Risk of Bias

Two reviewers independently assessed randomisation sequence generation, concealment of allocation, blinding of participants, RTW coordinators, and outcome assessors, completeness of data, whether participants were analysed in the group to which they were initially randomised, and whether selective outcome reporting occurred. Cluster RCTs were assessed for recruitment bias [23], and appropriate statistical analysis [23]. We assessed blinding of outcome assessment and completeness of data separately for RTW outcomes and patient reported outcomes (PROs). We used a modified Cochrane risk of bias instrument [23], with response options of “definitely yes”, “probably yes”, “probably no”, and “definitely no” with definitely and probably yes ultimately assigned high risk of bias and probably and definitely no assigned low risk of bias [24]. Because of the small number of studies for each outcome, we were unable to address publication bias or explore explanations for variability in results [23].

### Data Analysis

We conducted random effects meta-analyses (MAs) using RevMan 5.1 [25] and R 2.15.0 [26]. If available, we used baseline-adjusted effect estimates. In case of missing values, we analysed the available data without imputations to prevent biased weighting of studies [23]. We used I^2^ to estimate heterogeneity [23].

We expressed pooled effects of dichotomous outcomes as risk ratios and calculated illustrative absolute risk differences by using the median baseline risk. We pooled effects of continuous outcomes as differences between group means (mean differences).

We felt the most important outcome was RTW that persisted over the long term; if we found varying measures of RTW, we therefore focused on the one that best reflected long-term outcome. If studies with time to event outcomes failed to report hazard ratios (HR), we extracted individual patient data from survival curves, verified the extraction by re-plotting, and then calculated the HR and associated 95% confidence interval (CI). If data extraction was not possible, we calculated HRs and 95% CIs based on log-rank-tests [27].

Five reviewers independently grouped all PROs by consensus into 9 categories: Overall function, physical function, social function, mental function, general health, pain, depression, anxiety, and patient satisfaction. We preferred change scores to end scores in order to correct for possible baseline differences, but we pooled both types of scores as change scores were not available for all trials. We transformed PROs expressed in different units to units on the scale of the most familiar instrument before we pooled mean differences [28]. This allowed us to enhance the interpretation of the summary effect by considering an anchor based minimal important difference (MID) on that instrument. Specifically, we rescaled *overall function* into the 0 to 100 scale of the Oswestry Disability Index (MID = 10 [29]–[34]), *physical, mental and social function* into the 0 to 100 scale of the SF-36 (MIDs = 8.4, 7.3, and 11.7 [35], respectively) and *pain* into a 0 to 100 visual analogue scale (MID = 10 [36]). In a second step, we used the rescaled outcomes to calculate the proportion of participants who improved by at least one MID in each group of each trial which allowed us to calculate and pool risk differences (RD) [28].

We conducted sensitivity analysis if a study reported several definitions of a RTW-outcome, e.g. full-time and part-time RTW versus full-time only (specified in footnotes of table 3). If more than one study reported several definitions, we conducted meta-analyses of all possible combinations, that is six for *proportion at work at end of study* and six for *proportion ever returned to work*.

### Reporting and Rating Quality of Evidence

The PRISMA statement [37] guided our reporting and the GRADE framework [38] guided our assessment of confidence in the meta-analytic estimates.

## Results

### Identification of Eligible Trials and Data Collection

**Figure 1:**
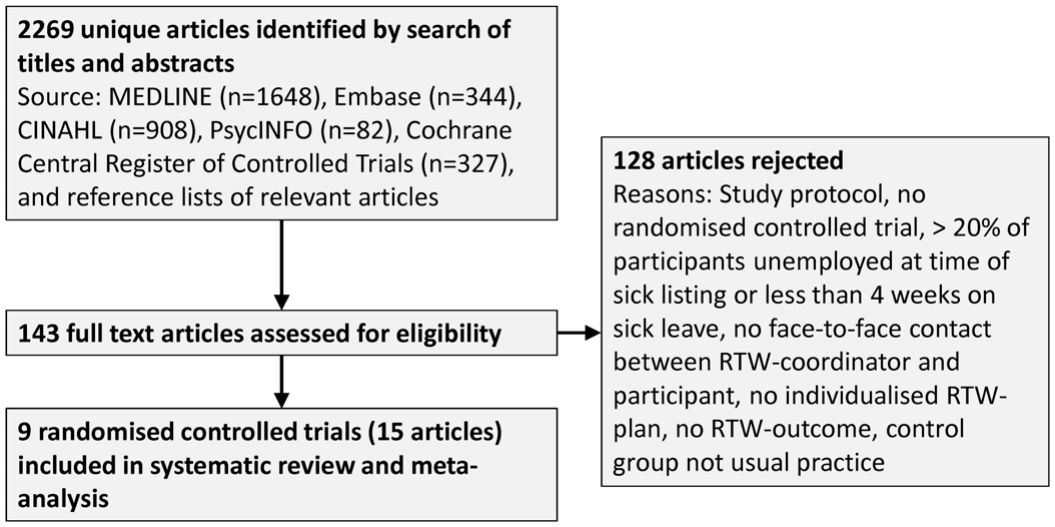
Study selection. Last update of electronic search to April 2, 2012.

Of 2459 citations, 15 articles [39]–[55] describing 9 RCTs proved eligible (figure 1). We approached 12 authors of whom 10 replied and 7 provided additional information about 7 studies [39]–[44], [46] (footnotes in tables 1, 2, 3, 4).

### Characteristics of Included Trials

Table 1 shows characteristics of studies and populations. Participants were consenting volunteers in all but one study in which participants received no official information about the intervention [46]. Table 2 shows characteristics of interventions and comparisons. No study specified the financial resources available to the RTW coordinators for patient support. In five studies [39], [40], [43], [45], [46], some participants assigned to practice as usual may have received RTW coordination.

**Table 1:** Characteristics of studies and populations (at time of randomisation).

Trial (country)	Sample size	Year(s), method of recruitment	Health condition	mean age (SD)[years]	% men	type of claim	length of work absence [months]
Bültmann 2009 (Denmark)	119	2004/2005, consecutive cases of sick leave registers of 4 municipalities	Musculoskeletal, not mental disorder	43.7 (11.3)	45	Full sick leave, not permanent disability	1 to 3
Davey 1994 (United Kingdom)	50	1990/1991, review of personal injury claimfiles of four participating personalinjury insurances	Injuries likely to result in absences fromwork of six months or more	39.4 (11.5)	75	Full sick-leave, not permanent disability	median 20 (range 3 to 55)
Donceel 1999 (Belgium)	710 in 60 clusters	1997/1997, consecutive cases of insuranceoffices ( = clusters)	Surgery for disc herniation	39.2 (n.r.)	65	Full sick leave	2 to 2.5
Feuerstein 2003 (Unites States of America)	205	1999/2000, claim database of the Departmentof Labour’s Office of Workers’ Compensation Programs	Work related upper extremity disorder	46.0 (8.6)^1^	22^1^	Full or part-time sick-leave	1 to 6^2^
Lambeek 2010 (Netherlands)	134	2005-2007, visitors of 4 outpatient clinics	Non-specific chronic low back pain, not mental disorder	46.2 (9.1)	58	Full or part-time sick leave, not permanent disability	3 or more
Lindh 1998 (Sweden)	611	1995/1996, consecutive cases of 7 socialinsurance offices	Non-specific chronic musculoskeletal pain	39.5 (n.r.)	38	Full sick-leave	3 or more
Purdon 2006 (United Kingdom)	1423	2003/2004, attracted by marketing of RTW-coordination-providers	Any condition likely to result in a<50% chance to returnto work without intervention, 1/3mental and 1/3 musculoskeletal disorders	44 (n.r.)	43	Full sick leave, not permanent disability	1,5 to 6
Rossignol 2000 (Canada)	110	1995/1996, consecutive casesof regional office of the Quebec Workers' CompensationBoard	Any work-related injury to the middle or lower vertebral column, not surgery or multiple injuries	37.6 (10.1)	72	Full sick-leave, not permanent disability	1 to 2
Van der Feltz-Cornelis 2010 (Netherlands)	60 in 24 clusters	2007, review of medical files and prospective selection by occupational physicians( = clusters)	Anxiety, depression, somatoform disorder	42, range 24–59 (n.r.)	42	Full sick leave, not permanent disability	mean 4.7, range 0.25 to 10.6

RTW = return to work, n.r. = not reported.

1 As inferred from a subsample of 131 participants.

2 From personal correspondence.

**Table 2:** Characteristics of interventions and comparisons.

Study: intervention title	Provider(s) of RTW-coordination:	Affiliation of RTW-coordinator(s)	Process of RTW-coordination	Duration	Consumption of health care and other services	Adherence of RTW-coordinators and participants	Usual practice
Bültmannn 2009: *“Coordinated and Tailored Work rehabilitation”*	1 rehab. team: OP, occupational PT, chiropractor, psychologist, social worker, experience and training n.r.	N.r.	Standardised assessment of disability and functioning, identification of barriers for RTW; individually tailored RTW-plan, actions directed at worker, workplace, and environment. Social worker coordinates with workplace and municipality case manager	Maximal 3 month	Increase	all patients received RTW-plan	Optional case management from municipal case managers
Davey 1994: “Rehabilitation co-ordinator service”	1 coordinator: PT, experience in care coordination, no specific training	Academic rehab. unit	Assessment at the participant's home, RTW-plan with focus on involving each claimant to the fullest possible extent, coordinator discussed plan with a psychologist and a physician, monitoring, making changes as appropriate	6 month	Increase	N.r.	No restriction
Donceel 1999: *“New guideline for medical advisers”*	30 medical advisers: social insurance physicians, experience and training n.r.	One private insurer	Monthly follow-up: Clinical and functional assessment, exploration of barriers for RTW, advice on legal criteria, gradual RTW, exercise, and normal course of work incapacity, encouragement of rehab., communication with treating physicians; case discussion with colleagues; referral to rehab. if no RTW after 3-4 months^3^	As long as participant on disability benefit	N.r.	no drop-outs^3^	30 medical advisors, focus on corporal damage, little rehab. efforts
Feuerstein 2003: “*Integrated case management*”	32 nurse case managers: 2 day training in ergonomic assessment and workplace accommodations, problem solving approach, experience in coordination of medical care	US Department of Labour	Semi structured interview, ergonomic worksite assessment, case management plan with workplace accommodation, applying problem solving process, monitoring, coordination of medical care (detailed list of workplace accommodations reported^2^)	4 month, variable	N.r.	N.r.	33 Nurse case managers, focus on medical care, no training in a structured protocol
Lambeek 2010: *“Integrated care”*	2 case managers: OPs, 2-day training program	University hospital	Individualised RTW-plan, coordination of care, communication with occupational therapists (mandatory workplace intervention based on participatory ergonomics) and physical therapists (mandatory graded activity program using cognitive behavioural principles). Conference calls every three weeks, strict timing.	67 (SD 32) calendar days	Decrease	N.r.	Guidance from OPs, GPs and other health professionals. averagely 0.2 visits to case managers
Lindh 1998: “*Multidisciplinary rehabilitation programme*”	1 rehab. team: rehab. physician, nurse, physical therapist, psychotherapist, psychologist, occupational therapist, social worker, vocational counsellor, experience and training n.r.	Outpatient rehab. clinic	Medical, functional, psychological and social assessment, RTW-plan, weekly team conferences, regular meetings with participant and spouse	Individually regulated	N.r.	N.r.	Physical therapy and other rehab. measures
Purdon 2006^1^: “*Job Retention and Rehabilitation*”	Case managers, experience and training n.r.	4 third-party case management providers	Point of contact for clients, giving advice, gate keeping to other services, sometimes providing services, coordination of medical care, rehab., employer, ergonomic workplace assessment, occupational therapy, advising on welfare rights, career, CV preparation, and job search.	20 to 36 weeks	No change	88% received RTW-plan, 72% of those followed the plan	No systematic aid; low levels of work support
Rossignol 2000: “P*rogram for coordination of primary health care* “	1 team: 2 primary care physicians, 1 nurse, experience and training n.r.	N.r.	Standardised medical assessment, RTW-plan according to clinical guideline for back pain. Assisting the treating physicians in finding and scheduling diagnostic and therapeutic procedures, cooperation with Worker's Compensation, standardised weekly telephone talk	Until RTW	No change	N.r.	Instruction to continue with treating physician
Van der Feltz-Cornelis 2010: “*psychiatric consultation model*”	12 OPs, training in diagnosis and treatment of mental disorders. consulted by 2 psychiatrist trained in improvement of work functioning	Company of participant	Psychiatric assessment, collaborative RTW-plan, coordination of plan and monitoring by OP	Until RTW	N.r.	N.r.	Care from OP^2^ and mental health care professionals

RTW = return to work, n.r. = not reported, OP = occupational physician, PT = physical therapist, GP = general practitioner, rehab. = rehabilitation.

1 The trial compared three intervention arms with usual practice. We considered only the arm “combined intervention” because the other arms were restricted to either workplace or health care interventions.

2 In the Dutch system, each company is obliged to have company insurance for sick leave and to offer their employees access to occupational health care. Occupational physicians provide social-medical guidance for sick listed employees with the aim to return to work (RTW) as quickly as possible. Usually, occupational physicians are organised as third party service providers.

3 From personal correspondence.

Table 3 shows details of the reported outcome measures. The outcome *proportion at work at end of study* best reflected long-term in contrast to *time until stable RTW* and *proportion ever returned to work* that provided information regarding the first episode of RTW or the first episode of RTW of a specific duration, and *sickness absence days* that expressed the duration of all episodes of sickness absence.

**Table 3:** Characteristics of outcomes.

Study	Follow-up	Return to work outcomes (definition); patient reported outcomes (assigned outcome group)
Bültmann 2009	12 months	**RTW** ^1^ **:** proportion at work at end of study^16^, sickness absence (mean number of work days off including all episodes of sick leave); **PROs** 17,18 **:** pain during last month (pain), Oswestry Low Back Pain Disability Questionnaire (general function); **Not analysed:** pain during last week (n.r.)
Davey 1994	6 months	**RTW** 2 **:** proportion at work (full or part time), proportion ever returned to work (full or part time); **PROs** 17 **:** Hospital Anxiety and Depression Scale^5^ (depression), Nottingham Health Profile (physical and social function)^5^; **Not analysed:** self-rated anxiety
Donceel 1999	12 months	**RTW** 4 **:** proportion at work at end of study (full time) = proportion ever returned to work, time until RTW^6^ (full-time, “stable” at end of follow up)
Feuerstein 2003	16 months	**RTW** ^1^: time until RTW^7,8,9,10, 11^ (full time), proportion ever returned to work (full time) 8,10, 11; **PROs** 17 **:** Patient satisfaction, Upper extremity function scale^7^ (pain), SF-12^7^ (physical and mental function); **Not analysed:** Levine symptom scale (overlap with other functional scales)
Lambeek 2010	12 months	**RTW** ^3^ **:** time until RTW (full-time, for at least 4 weeks), proportion ever returned to work^8^, sickness absence (mean number of work days off including all episodes of sick leave); **PROs:** Roland disability questionnaire (physical function)^18^, visual analogue scale (pain)^18^, EQ5D (general function)^7,17^
Lindh 1998	60 months^12^	**RTW** ^1^ **:** proportion at work at end of study^8,10,12, 13^ (full or part time), proportion ever returned to work^8,10,12,13^ (full or part time)
Purdon 2006	20 to 36 weeks	**RTW** 2 **:** proportion at work at end of study, proportion ever returned to work (full-time, for at least 2 weeks^14^); **PROs** 17 **:** SF-36 (physical, mental and social function, pain, general health); **Not analysed:** Hospital Anxiety and Depression Scale^15^, cumulative sickness absence^15^, cumulative Incidence of RTW for a spell of 13 weeks^14^; SF-36 (physical role, emotional role, energy/fatigue), self-assessed general health, health improvement
Rossignol 2000	6 months	**RTW** ^1^ **:** time until RTW (full or part time, for at least 2 days)^11^; **PROs** 18 **:** visual analogue scale (pain), Quebec Back Pain Disability Scale (physical function), Oswestry low back pain disability questionnaire (overall function); **Not analysed:** Dallas pain questionnaire (overlap with other functional scales), Health care satisfaction (n.r.)
Van der Feltz-Cornelis 2010	6 months	**RTW** ^3^ **:** time until RTW (full-time, for at least 4 weeks, all who returned stayed at work)^8,10,13^, proportion at work at end of study^10,13^ = proportion ever returned to work^10,13^; **PROs** 18 **:** PHQ9 (depression); **Not analysed:** PHQ15, SCL-90 (no subscales reported), EQ5D (reported in Quality Adjusted Life Years)

RTW = return to work, PROs = patient reported outcomes, n.r. = not reported.

Data source: ^1^administrative data,

2 diary, interview, or survey, ^3^combination of diary or interview and administrative data,

4 not reported.

5 SD not reported. We imputed missing standard deviations (SDs) with the weighted average of the SDs of the remaining trials.

6 Hazard ratio estimated from log-rank test.

7 From personal correspondence.

8 Data extracted from graph.

9 Time between a claimant’s initial evaluation by a case manager (not randomization) and RTW.

10 Missing or unclear number of participants.

11 Cessation of disability benefits as surrogate for RTW (Rossignol, Feuerstein).

12 Data presented for two subgroups (immigrant and swedes) which we recombined. Only the number of patients who started the intervention reported. To prevent attrition bias at 60 month, we used 18 month data (ensuring a slightly longer follow up than other studies) and conducted sensitivity analysis using 15 or 12 months.

13 Data in graph conflicting with text or table.

14 RTW for at least 2, 6 or 13 weeks reported. We disregarded the 13 weeks outcome which most participants could not achieve due to short follow-up. To ensure longest follow-up, we used 2 weeks and conducted sensitivity analysis using 6 weeks.

15 Data presented in groups, variance not estimable.

16 Full-time and part-time RTW reported separately. We used full time RTW and conducted sensitivity analyses using part-time combined with full-time.

17 End scores.

18 Change scores.

### Risk of Bias

Table 4 presents our assessment of risk of bias. See footnotes of table 4 for unclear or incomplete reporting of outcomes that we could not clarify with authors. Most studies concealed allocation and conducted an analysis-as-randomised. Blinding of personnel, participants and assessors of patient reported outcomes (self-administered questionnaires) was impossible. Loss to follow-up was substantial in most studies.

**Table 4:** Methodological components.

Study	Random sequence adequately generated?	Allocation concealed?	Participants and RTW-coordinators blinded?	RTW-outcome assessor blinded?	PRO- outcome assessor blinded?	Loss to follow-up of RTW-outcomes [%]	Loss to follow-up of PROs [%]	Intention to treat analysis^1^	Selective reporting^2^	Other
Bültmann 2009	Y	Y^3^	N	Y	N	5	34	Y	?	
Davey 1994	Y	Y	N	N	N	0	0	Y	?	
Donceel 1999	Y	Y	N^4^	N	n.a.	0	n.a.	(Y)	?	9
Feuerstein 2003	Y	N^3^	N	(N)	N	40^3^	36–61	(N)	Y^5,8^	
Lambeek 2010	Y	Y	N	N	N	7	13	Y	N	
Lindh 1998	(N)	(N)	N	Y	n.a.	?	n.a.	Y	Y^6,8^	
Purdon 2006	Y	Y^3^	N	N	N	28	29	Y	?	
Rossignol 2000	Y	Y	N	Y	N	0	18	Y	?	
V. d. Feltz-Cornelis 2010	Y	Y	N	Y	N	18	27	Y	Y^7,8^	9

RTW = return to work, PRO = patient reported outcomes, Y = yes, (Y) = probably Yes, N = No, (N) = probably no, ? = unclear, n.a. = not applicable.

1 Participants analysed in the group to which they were initially assigned.

2 “No” if protocol published and all outcomes correctly reported; “?” if no protocol published and selective reporting not obvious.

3 From personal correspondence.

4 Participants were probably not aware of the intervention.

5 RTW-outcomes not published, incomplete outcome information (see table 3).

6 Results presented in subgroups, incomplete outcome information (see table 3).

7 Primary outcome not mentioned in protocol.

8 Incomplete outcome information (see table 3).

9 Cluster randomised trials: No risk of recruitment bias. Baseline information of individual clusters not reported. Effects of RTW-outcomes not corrected for possible design effects (risk of inflated precision).

### Effects and Confidence in Estimates

Table 5 shows the evidence profile of the meta-analytic estimates of important outcomes and Table S1 the summary of findings table for all outcomes. The heterogeneity was low across all outcomes but risk of bias (high attrition or selective reporting), imprecision and indirectness limited our confidence in the estimates.

**Table 5:** Evidence Profile, relevant outcomes.

Quality assessment						No of participants		Effect			Confidence in estimate		Importance
No of studies	Design	Risk of bias	Inconsistency	Indirectness	Imprecision	RTW-coordination	Usual care	Relative (95% CI)	Absolute				
**Proportion at work et end of study**													
6	randomised trials	serious^1,2^	no serious inconsistency	no serious indirectness	no serious imprecision	794/1279 (62.1%)	656/1138 (57.6%)	RR 1.08 (1.03 to 1.13)	5 more per 100(from 2 more to 7 more)		⊕⊕⊕ MODERATE		CRITICAL
**Overall function (range of scores: 0-100; Better indicated by higher values)**													
4	randomised trials	serious^1^	no serious inconsistency	no serious indirectness	no serious imprecision	716	558	–	MD 5.2 higher(2.4 to 8.0 higher)	⊕⊕⊕ MODERATE			IMPORTANT
**Physical function (range of scores: 0-100; Better indicated by higher values)**													
5	randomised trials	serious^1^	no serious inconsistency	no serious indirectness	no serious imprecision	729	619	–	MD 5.3 higher(1.4 to 9.1 higher)			⊕⊕⊕ MODERATE	IMPORTANT
**Pain (range of scores: 0-100; Better indicated by lower values)**													
6	randomised trials	serious^1,4^	no serious inconsistency	no serious indirectness	no serious imprecision	784	646	–	MD 6.1 lower(3.1 to 9.2 lower)			⊕⊕⊕ MODERATE	IMPORTANT
**Social function (range of scores: 0-100; Better indicated by higher values)**													
2	randomised trials	serious^1^	no serious inconsistency	no serious indirectness	serious^5^	589	470	–	MD 3.1 higher (0.6 lower to 6.8 higher)			⊕⊕ LOW	IMPORTANT
**Mental function (range of scores: 0-100; Better indicated by higher values)**													
2	randomised trials	serious^1,2^	no serious inconsistency	no serious indirectness	no serious imprecision	599	512	–	MD 3.1 higher (0.7 to 5.6higher)			⊕⊕⊕ MODERATE	IMPORTANT

1 Risk of attrition bias.

2 Risk of reporting bias.

3 Total population size less than 400.

4 Use of unvalidated instruments.

5 Confidence interval encloses no effect and meaningful difference.

**Figure 2:**
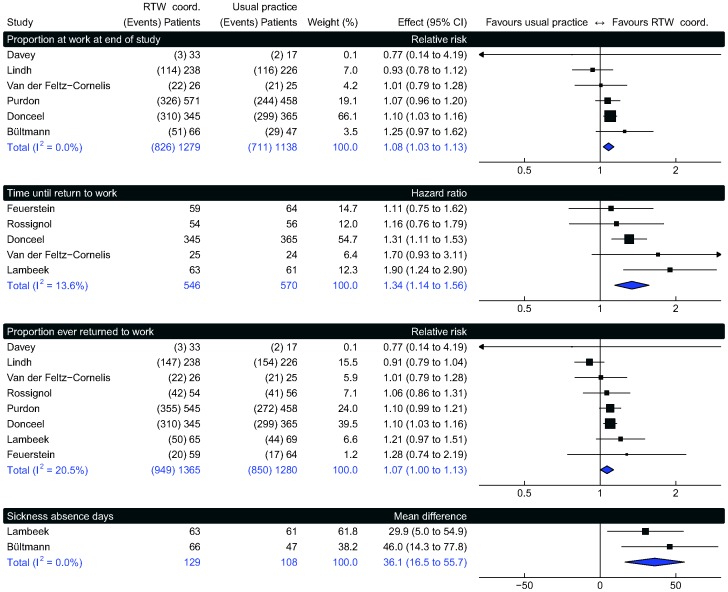
RTW-outcomes. RTW coord. = return to work coordination.

All pooled effects of RTW outcomes significantly favoured RTW coordination (figure 2). The *proportion at work at end of study* increased by a factor of 1.08 (95% confidence interval (CI) 1.03 to 1.13, moderate confidence). This corresponds to an absolute effect of 5 in 100 more individuals returning to work (95% CI 2 more to 8 more). The pooled hazard ratio of *time until stable RTW* was 1.34 (95% CI 1.12 to 1.36, moderate confidence). The *proportion of ever returning to work* increased by a factor of 1.07 (95% CI 1.00 to 1.13, low confidence), corresponding to 4 more per 100 (95% CI, 0 more to 8 more). Total s*ickness absence days* decreased by 36 workdays per year (95% CI, 17 to 56, moderate confidence). Sensitivity analysis did not reveal any substantial differences in our pooled estimates or heterogeneity.

**Figure 3:**
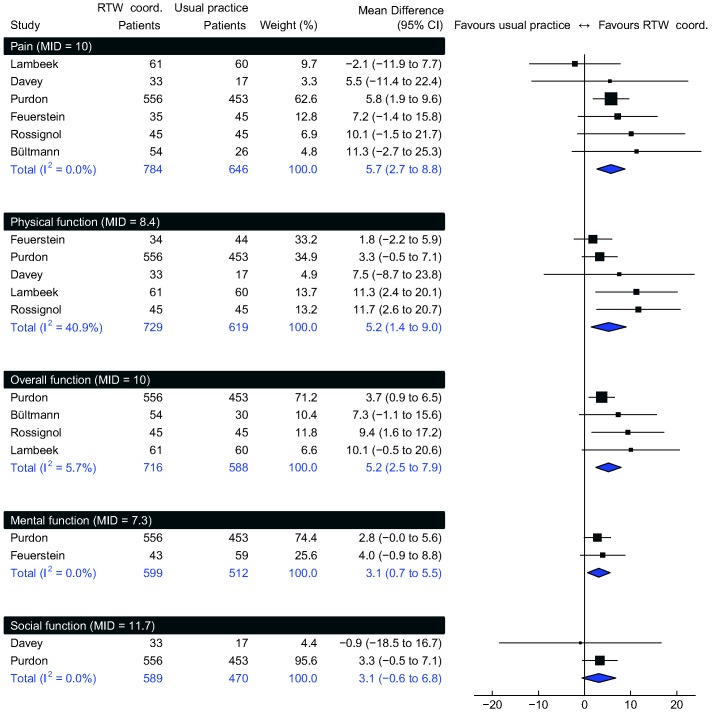
Patient reported outcomes. Individual trials’ outcomes expressed on a 0 to 100 scale. RTW coord. = return to work coordination. MID = minimal important difference.

Figure 3 shows meta-analyses of PROs. Expressed on a 0 to 100 scale, RTW coordination improved *mean overall function* by 5.2 (95% CI 2.4 to 8.0; MID = 10, moderate confidence), *physical function* by 5.3 (95% CI 1.4 to 9.1; MID = 8.4, moderate confidence), *pain* by 6.1 (95% CI 3.1 to 9.2; MID = 10, moderate confidence), *mental function* by 3.1 (95% CI 0.7 to 5.6; MID = 7.3, moderate confidence) and *social function* by 3.1 (95% CI –0.6 to 6.8; MID = 11.7, low confidence). When we used the MIDs to calculate risk differences, RTW coordination increased the proportion of participants who improved considerably in *overall function* by 9% (95% CI 4 to 15%), *physical function* by 8% (95% CI 2 to 14%), *pain* by 8% (95% CI 2 to 13%), *mental function* by 6% (95% CI 0 to 11%), and *social function* by 4% (95% CI –2 to 10%).

Figure S1 shows the output of the RevMan software including the raw data.

## Discussion

We found moderate quality evidence that RTW coordination interventions result in small relative increases in RTW. Assuming a typical risk of 43 in 100 individuals not returning to work, this small relative effect implies an absolute effect of 5 in 100 more returning to work. If maintained over the long term, many would consider this an important benefit. We also found moderate quality evidence that the intervention results in small improvements in function and pain. We found no evidence that one type of RTW coordination programme was superior to another.

Our findings gain credence from the rigor of the review. We performed a comprehensive search, adjudicated eligibility and extracted data independently and in duplicate, obtained additional information from 7 authors, performed appropriate primary and sensitivity analyses and evaluated confidence in estimates of effect using the GRADE approach [38].

Our review has limitations. First, given the small number of studies for each outcome, we were unable to address publication bias. Second, we pooled change and end scores for the PROs. In theory, standard deviations of the two scores might differ substantially, leading to different weighting of individual studies in the meta-analysis [23]. However, there is evidence that SDs of change scores often do not appreciably differ from end scores [56]. Third, results from two cluster RCTs uncorrected for intra-cluster dependency may have spuriously increased precision, thus overweighting these studies in the meta-analysis.

### Comparison with Other Systematic Reviews

Our study selection partly overlaps with related systematic reviews that defined RTW interventions from different points of view. They compared usual practice to RTW interventions that either included a specific workplace component [12]–[15], applied RTW-interventions to a population with a specific health condition [16]–[19], or explored them within a specific country only [20]–[22]. Two of these systematic reviews (with 3/42 [17] or 0/10 [13] studies overlapping) addressed RTW coordination in a subgroup analysis (RTW coordination as a subgroup of RTW-interventions). Both suggested that RTW coordination improved RTW [13], [17] whereas effects on PROs remained unclear [13]. However, much like other related reviews, they did not perform a meta-analysis. Reasons included poor study quality [15] or high heterogeneity in the RTW interventions [15], [17], [18]. Only one systematic review (1/6 studies overlapping) conducted a meta-analysis, concluding with low confidence that RTW interventions with an active workplace involvement improve RTW outcomes [12].

Other reviews also noted limitations in the evidence that we identified. Evidence regarding the effectiveness of RTW interventions suffers from poor descriptions of interventions and controls [12], [13], insufficient information beyond one year follow-up [13], [18], and paucity of studies on participants with mental health problems [12], [13]. Further, a systematic review of 34 RCTs (3 overlapping) and 8 cohort studies found evidence of possible publication bias [17].

### Applicability of Findings

Applicability of the results is enhanced by recruitment through insurance registers that ensured a representative selection of claimants. The prompt initiation of interventions after work absence and the high intensity of support are consistent with the OECD recommendations that social insurances or corresponding benefit authorities should apply RTW coordination at an early stage and resources should shift from passive benefits towards RTW programmes [1].

Diversity and limitations in the description of both RTW coordination interventions, and the nature of usual practice, advise on cautious interpretation and application of our results. Most studies focused on organisational features, such as composition of the team, distribution of roles, and standardisation of initial assessment. Interventions differed in degree of standardisation, and in the roles and backgrounds of intervention providers. Information regarding training and experience of RTW coordinators, resources available, and adherence of coordinators and participants were typically lacking. Descriptions of the usual practice controls were even more limited.

The striking consistency of results from study to study in virtually all outcomes ameliorated the unease about variability in interventions and controls. If variability were very important, one would not expect to see such consistency.

All but 2 studies [42], [45] (85% of participants in the review) focused on claimants with musculoskeletal complaints. Recent statistics from high-income countries show that new disability claimants with psychiatric disorders (30 to 40%) have outnumbered those with musculoskeletal complaints [1]. Although the results from the two studies that did enrol a substantial proportion [42] or an exclusive sample [45] of claimants with psychiatric complaints showed similar results to other studies, generalizing results to these populations is questionable.

Judging the importance of our measured relative effect size is challenging. An absolute difference in the proportion at work at end of study - of the order of 5% suggested by the results of this review - could be important if maintained over the long term. Indeed, many are likely to agree that an absolute reduction in the proportion on long-term disability would be important. However, follow-up was generally too short to inform results over the long-term. Only one study assessed work stability after initial work resumption but reported the results incompletely [47].

Two studies conducted an economic analyses based on the outcome *cumulative sickness absence* [39], [50] one year after randomisation. They both concluded that RTW coordination compared to usual practice was cost effective from a societal perspective, that is by considering the cost of the intervention, health care utilisation, and loss of productivity. The societal perspective leaves out the cost of wage replacement, which is considered a redistribution of wealth, and, therefore, does not inform about the impact of RTW coordination on social security savings. In contrast, an economic analysis from an insurance perspective would integrate this information. Cost effectiveness from an insurance perspective may occur only in the long-term and depend mainly on savings related to fewer disability pensions [57].

### Implications for Research

Results to date suggest small but possibly important benefits of RTW coordination. Determining the long-term benefits and the cost effectiveness of the programmes will require trials with low risk of bias (concealment, blinding of outcome assessors and statisticians, minimal missing data), measuring long-term outcomes of work force retention and long-term disability (including pensions). This would also enable extending the research on comparing different definitions of RTW outcomes [58]. We require studies in specific populations that represent the majority of disabled individuals, including both musculoskeletal and psychiatric problems. We strongly encourage researchers of RTW interventions to describe interventions, comparisons, and settings more systematically to enable comparability of studies and facilitate transfer into practice.

## Supporting Information

Figure S1 RevMan output for all outcomes including raw data.
(DOCX)

Table S1 Summary of findings for all outcomes.
(DOCX)

Document S1 Protocol.
(DOCX)

Document S2 PubMed search strategy.
(DOCX)
