# Supplementary material for: Return to Work Coordination Programmes for Work Disability: A Meta-Analysis of Randomised Controlled Trials
Source: PLoS One. 2012 Nov 19;7(11):e49760. doi: 10.1371/journal.pone.0049760 (PMC3501468; doi:10.1371/journal.pone.0049760)

# Figure S1, RevMan output including raw data

## Proportion at work at end of study


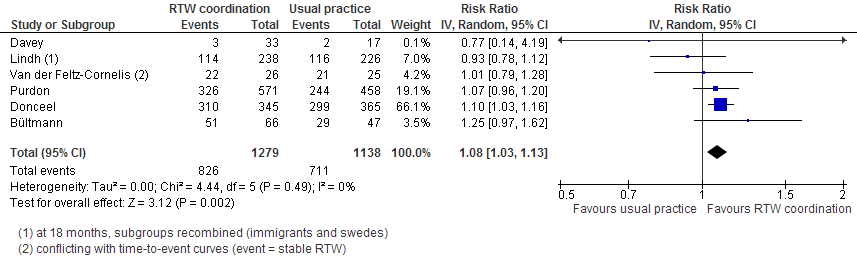


## Time until RTW


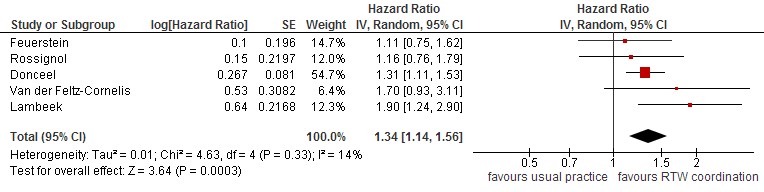


## Proportion ever returned to work


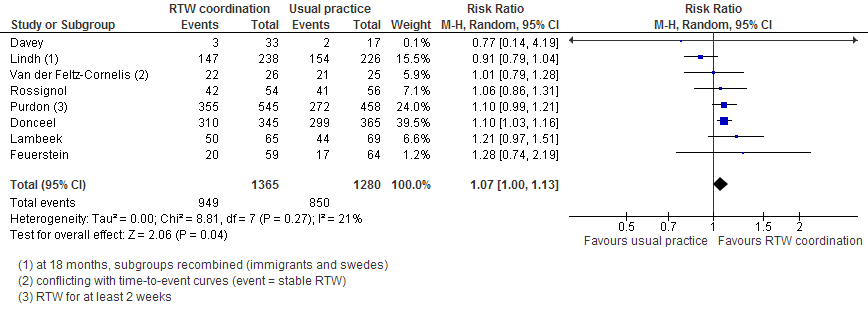


## Sickness absence


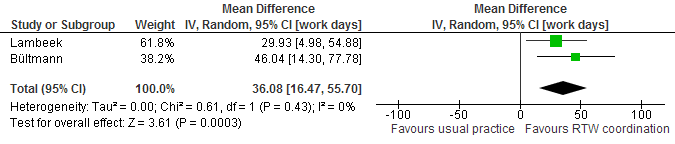


## Overall function


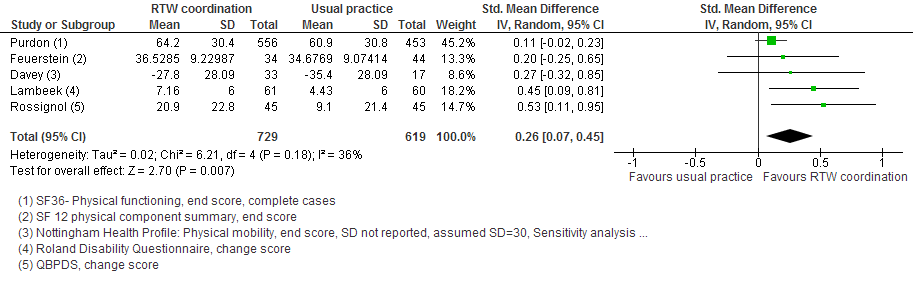


## Physical function


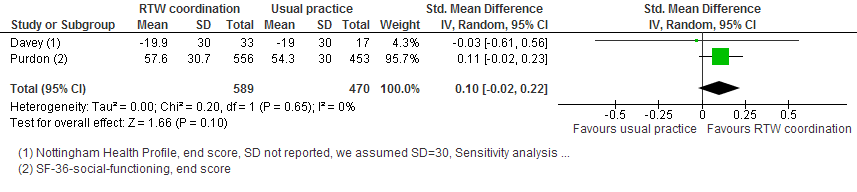


## Pain


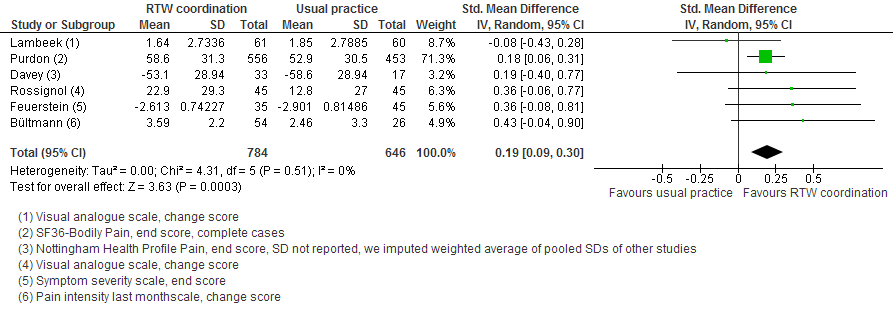


## Social function


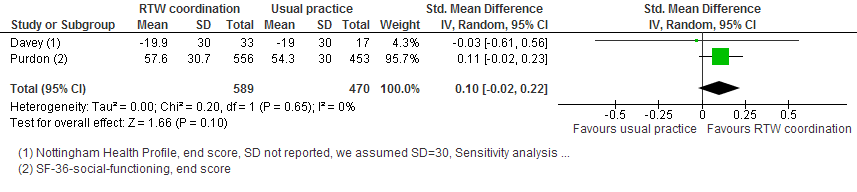


## Mental function


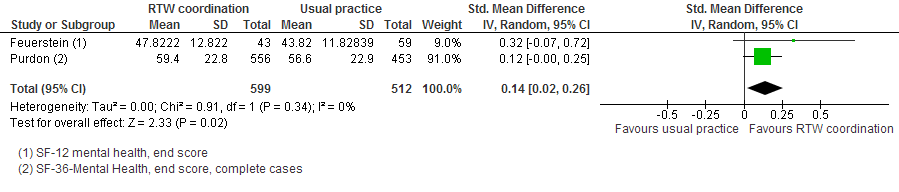


## Depression


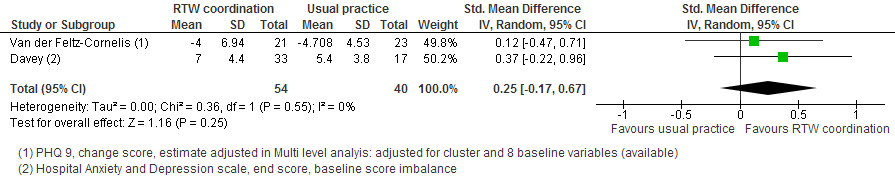


## Anxiety


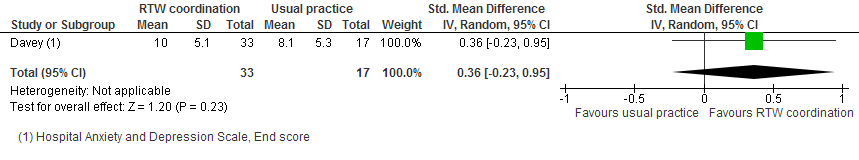


## Patient Satisfaction


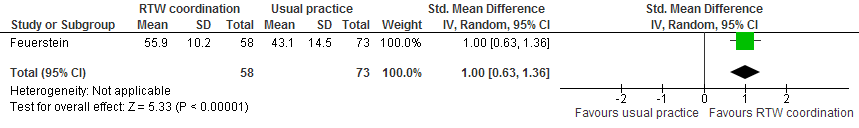

Supplement: Figure S1 — RevMan output for all outcomes including raw data. (DOCX) [file pone.0049760.s001.docx]
