# Supplementary material for: Return to Work Coordination Programmes for Work Disability: A Meta-Analysis of Randomised Controlled Trials
Source: PLoS One. 2012 Nov 19;7(11):e49760. doi: 10.1371/journal.pone.0049760 (PMC3501468; doi:10.1371/journal.pone.0049760)
Supplement: Table S1 — Summary of findings for all outcomes. (DOCX) [file pone.0049760.s002.docx]

**Table S1, summary of findings, all outcomes**

| **Outcomes** | **Illustrative comparative risks* (95% CI)** | | **Relative effect (95% CI)** | **No of Participants (studies)** | **Confidence in the estimate (GRADE)** |
| --- | --- | --- | --- | --- | --- |
|  | Assumed risk | Corresponding risk |  |  |  |
|  | **Usual care** | **RTW-coordination** |  |  |  |
| **Proportion at work et end of study** | **58 per 100** | **62 per 100** (59 to 65) | **RR 1.08**  (1.03 to 1.13) | 2417 (6 studies) | ⊕⊕⊕⊝ **moderate**^1,2,3^ |
| **Time until RTW** |  |  | **HR 1.34**  (1.14 to 1.56) | 1116 (5 studies) | ⊕⊕⊕⊝ **moderate**^4^ |
| **Proportion ever returned to work** | **64 per 100** | **70 per 100** (64 to 76) | **RR 1.07**  (1.00 to 1.13) | 2645 (8 studies) | ⊕⊕⊝⊝ **low**^1,2,4^ |
| **Sickness absence** | The mean sickness absence in the control groups was **109.1 work days during one year** | The mean sickness absence in the intervention groups was **36.1 higher** (16.5 to 55.7 higher) |  | 237 (2 studies) | ⊕⊕⊕⊝ **moderate**^3^ |
| **Overall function**  Scale from: 0 to 100. |  | The mean overall function in the intervention groups was **5.2 higher** (2.4 to 8.0 higher) |  | 1274 (4 studies) | ⊕⊕⊕⊝ **moderate**^1^ |
| **Physical function** Scale from: 0 to 100. |  | The mean physical function in the intervention groups was **5.3 higher** (1.4 to 9.1 higher) |  | 1348 (5 studies) | ⊕⊕⊕⊝ **moderate**^1^ |
| **Pain** Scale from: 0 to 100. |  | The mean pain in the intervention groups was **6.1 lower** (3.1 to 9.2 lower) |  | 1430 (6 studies) | ⊕⊕⊕⊝ **moderate**^1,5^ |
| **Social function** Scale from: 0 to 100. |  | The mean social function in the intervention groups was **3.1 higher** (0.6 lower to 6.8 higher) |  | 1059 (2 studies) | ⊕⊕⊝⊝ **low**^1,6^ |
| **Mental function** Scale from: 0 to 100. |  | The mean mental function in the intervention groups was **3.1 higher** (0.7 to 5.6 higher) |  | 1111 (2 studies) | ⊕⊕⊕⊝ **moderate**^1,2^ |
| **Depression** |  | The mean depression in the intervention groups was **0.25 standard deviations lower** (0.67 lower to 0.17 higher) |  | 94 (2 studies) | ⊕⊕⊝⊝ **low**^1,2,3,6^ |
| **Anxiety** |  | The mean anxiety in the intervention groups was **0.36 standard deviations lower** (0.95 lower to 0.23 higher) |  | 50 (1 study) | ⊕⊕⊝⊝ **low**^1,3,6^ |
| **Patient Satisfaction** |  | The mean patient satisfaction in the intervention groups was **1.00 standard deviations higher** (0.63 to 1.36 higher) |  | 131 (1 study) | ⊕⊝⊝⊝ **very low**^1,2,3,5^ |
| *The basis for the **assumed risk** was the the median control group risk across studies. The **corresponding risk** (and its 95% confidence interval) is based on the assumed risk in the comparison group and the **relative effect** of the intervention (and its 95% CI). **CI:** Confidence interval; **RR:** Risk ratio; **HR:** Hazard ratio; | | | | | |
| GRADE Working Group grades of evidence **High quality:** Further research is very unlikely to change our confidence in the estimate of effect.  **Moderate quality:** Further research is likely to have an important impact on our confidence in the estimate of effect and may change the estimate. **Low quality:** Further research is very likely to have an important impact on our confidence in the estimate of effect and is likely to change the estimate. **Very low quality:** We are very uncertain about the estimate. | | | | | |
| ^1^ Risk of attrition bias  ^2^ Risk of reporting bias ^3^ Imprecision: total population size less than 400 ^4^ Indirectness: the studies inferred stable RTW from short term, that is, the definition of stable RTW required only a short duration of continued work or was limited by duration of study. ^5^ Use of unvalidated patient-reported outcomes ^6^ Imprecision: confidence interval encloses no effect and meaningful difference | | | | | |
