# Supplementary material for: Return to Work Coordination Programmes for Work Disability: A Meta-Analysis of Randomised Controlled Trials
Source: PLoS One. 2012 Nov 19;7(11):e49760. doi: 10.1371/journal.pone.0049760 (PMC3501468; doi:10.1371/journal.pone.0049760)
Supplement: Document S1 — Protocol. (DOCX) [file pone.0049760.s003.docx]

The Effectiveness of return to work coordination programs for work disability: a systematic review of randomised controlled trials

Protocol (version 4.0 – 2010-12-08)

# Study Team:

Stefan Schandelmaier, Shanil Ebrahim, Susan CA Burkhardt, Wout EL de Boer, Thomas Zumbrunn, Gordon H Guyatt, Jason W Busse, Regina Kunz

Study Objective

To determine the effectiveness of return to work (RTW) coordination compared to practice as usual in patients on work disability for at least 4 weeks on permanent disability, RTW, functioning, and quality of life.

# Criteria for considering studies for this review

Type of studies

Randomized controlled trials

Type of participants

People in working age (16-65 yrs.) who

1. have been on full or part time sick leave continuously for at least 4 weeks prior to randomisation irrespective of the health problem (mental and musculoskeletal complaints, or any other health condition) OR receive permanent disability benefits
2. have been employed at the time of sick-listing

80% or more of participants in a study have to match both criteria defined under a) and b).

NB: Schemes to cover sick-leave or disability vary within Europe and between Europe and North America. For instance, in Europe, the payment for sick leave usually lasts between 6 months to 2 years, followed by disability benefit. In the US/Canada, sick leave usually lasts between 4 to 6 weeks, followed by a 6 month period of short term disability benefit, followed by long-term disability benefit. We include studies independently of the benefit scheme.

We do **not** consider trials on participants suffering from birth defects.

We do **not** consider interventions that focus on primary prevention of sick-leave

Type of interventions

Given the lack of a uniform definition for RTW coordination and a variety of synonyms (e.g. case management, integrated or collaborative care, and multidisciplinary rehabilitation), we define RTW coordination in the context of this review as follows:

1. Objective RTW
2. Includes at least one face-to-face contact between the RTW coordinator(s) and the affected individual
3. Process starts with an assessment of the client’s needs and leads to an individually tailored RTW-plan.
4. The RTW coordinator/ the RTW coordination team coordinates the implementation of the plan

"Individually tailored" in this context stands for an individually compiled set of actions directed at the worker, the employer, the workplace, or other factors in the RTW process. It does not stand for an individual adjustment within an action such as individually tailored physical therapy. This also implies that the RTW coordination-intervention has to allow for more than one possible action.

Type of comparison

We compare RTW coordination Intervention to *practice as usual* that is the current standard as described by the study authors.

"Practice as usual" may range from "no RTW coordination" (such as "general practitioner care without additional support from a social insurance organisation or an authority) to "other RTW coordination".

Setting

Our aim is to look at studies where public or private insurers of income offer RTW coordination to people on work absence. In addition, we will consider a setting "insurance-like" when the RTW coordination intervention is a kind of service that could be contracted by a social insurance organisation. We assume that RTW coordination in an insurance-like setting has comparable effects to RTW coordination offered by a social insurance organisation as long as both share the goal RTW.

Outcome measures

Our primary outcome is disability status or RTW. Since a uniform agreed definition of RTW is lacking we will all definitions of study authors. We exclude a study if no such outcome is reported.

Definitions of primary outcomes may include

- proportion receiving permanent disability pension
- proportion at work at a specific time point
- Time until lasting RTW (requires a definition of "lasting RTW" such as RTW for at least four weeks at a time without recurrent work absence).
- Time until first RTW
- Cumulative sickness absence during follow up
- Cumulative sickness absence after RTW
- Time until recurrence of sick leave *after* initial RTW

The definition of RTW may further include whether the individual returns to

- the former or a new employer
- the former, a modified, or a new occupation

We will also collect secondary, patient reported outcomes including

- Functioning such as physical, mental, or social functioning
- Quality of life
- Health status such as pain or depression
- Satisfaction of patients, employers, and social insurance organisations

# Literature Search:

We will search the following databases: PubMed (including the "related citations" feature); EMBASE; Cochrane Central Register of Controlled Trials, CINAHL; PsycINFO.

We will develop a search strategy with terms designed to capture patients who are absent from work and RTW coordination interventions, and a filter for randomised controlled trials.

Reviewers will scan the bibliographies of all retrieved trials and other relevant publications, including reviews and meta-analyses, for additional relevant articles. For those relevant publications that are listed in the ISI Web of Science we will scan citing articles with the "times cited" feature.

# Selection of studies

Two reviewers will independently review the title and abstracts of the identified citations and collect the full text of all studies that at least one reviewer deems potentially eligible. In a second step, the 2 reviewers assess the full publication for eligibility and compare the results. Disagreement will be discussed, if necessary, with a third reviewer.

# Data abstraction

Data abstraction forms will be designed and piloted for a comprehensive assessment of characteristics of patients, interventions and comparisons (providers, features, process); primary and secondary outcomes, setting, and study methodology. Abstracted data will include the following variables:

*Demographic and Related Characteristic*

Age, Gender, Duration of sick leave, Level of education, Occupational status, Involvement in litigation, Clinical condition, medical or psychiatric co-morbidities listed as exclusion criteria

*Interventions and comparisons*

Individual RTW coordinator or RTW coordination team, professional background(s) of RTW coordinator(s), training of RTW coordinator(s), funding of intervention, financial resources available to the RTW coordinators for client support, time frame and time schedule of intervention, frequency of contact RTW coordinator / patient, specific features of RTW coordination beside eligibility criteria, concomitant interventions

*Outcomes*

All reported outcomes of interest.

Two reviewers will independently complete the data extraction forms and resolve disagreement by discussion. We attempt to contact study authors to resolve any uncertainties.

# ****Assessment of Risk of bias****

We will use the tool of the Cochrane Collaboration for assessing risk of bias. [1]

# Data analysis

If possible, we will calculate random effects meta-analyses using Review Manager. [2]

We will express dichotomous outcomes as relative risks and time to event outcomes as hazard ratios.

We will use minimal important differences to improve interpretation of continuous outcomes.[3]

We will assess heterogeneity using I^2^ statistics. In case of substantial heterogeneity and in case a meta-analysis will include at least ten studies, we will conduct subgroup analysis. We conducted a DELPHI consensus among 5 reviewers to prespecify 5 hypotheses for possible subgroup differences.

1. Trials with high risk of bias show larger effects
2. Populations with longer history of sick leave (length of continuous sick leave, number/length episodes in the past year) show smaller effects
3. Effects increase with Intensity of RTW coordination (Case load of RTW coordinators, patient participation, Frequency of contact, length of intervention phase)
4. Effects decrease with Intensity of support in the control group (ranging from no support to other form of RTW coordination)
5. Effects increase with involvement of employers

If a meta-analysis will include 10 studies or more we will explore possible publication bias with funnel plots and test for funnel plot asymmetry [1].

# Interpretation and presentation of results

We will use the GRADE methodology to assess our confidence in each meta-analytic estimate and present the results. [4] Specifically, GRADE will guide our

1. assessment of our confidence in estimates integrating study limitations, inconsistency, indirectness, imprecision, and publication bias.
2. reporting of results in the structured format of “Summary of Findings" and "Evidence Profiles" tables, where each relevant outcome will be reported separately together with illustrative absolute effects.

# Amendments to the protocol:

1. We withdrew the eligibility criterion “*prospective non-randomized controlled trials*” because we found enough RCTs (amendment: 2010/10)
2. We withdrew the eligibility criterion “minimum follow up of 3 month after RTW in > 80% of cases" since none of the potentially eligible studies fulfilled this criterion
3. We added the exclusion criterion: “We exclude trials if more than 20% of the participants were unemployed at the time of sick listing” (*2010/9/20*)
4. We refined the description of setting *(2010/10/1)*
5. We refined the description of setting *(2010/12/28)*
6. We added the exlusion criterion: no RTW-outcome reported. *(2010/12/28)*
7. We added a note defining "individually tailored" *(2010/12/28)*

# References

1. Higgins JPT, Green S, editors (2008) Cochrane Handbook for Systematic Reviews of Interventions Version 5.0.0 [updated February 2008]. The Cochrane Collaboration. p. Available:www.cochrane-handbook.org.

2. Review Manager (RevMan) (2008). Copenhagen: The Nordic Cochrane Centre, The Cochrane Collaboration. p. Available:http://ims.cochrane.org/revman/.

3. Johnston BC, Thorlund K, Schünemann HJ, Xie F, Murad MH, et al. (2010) Improving the interpretation of quality of life evidence in meta-analyses: the application of minimal important difference units. Health and quality of life outcomes 8: 116. doi:10.1186/1477-7525-8-116.

4. Guyatt GH, Oxman AD, Vist GE, Kunz R, Falck-Ytter Y, et al. (2008) GRADE: an emerging consensus on rating quality of evidence and strength of recommendations. BMJ 336: 924–926. doi:10.1136/bmj.39489.470347.AD.
