# Supplementary material for: Return to Work Coordination Programmes for Work Disability: A Meta-Analysis of Randomised Controlled Trials
Source: PLoS One. 2012 Nov 19;7(11):e49760. doi: 10.1371/journal.pone.0049760 (PMC3501468; doi:10.1371/journal.pone.0049760)
Supplement: Document S2 — PubMed search strategy. (DOCX) [file pone.0049760.s004.docx]

# Document S 2, PubMed Search Strategy

1. "absenteeism"[tw]

2. "sick days"[tw]

3. "illness days"[tw]

4. "sick listed"[tw]

5. **(**"long term"[tw] OR "absence"[tw] OR "leave"[tw]**) AND (**"disability"[tw] OR "sickness"[tw] OR "sick"[tw]**)**

6. ("benefit"[tw] OR "benefits"[tw] OR "pension"[tw] OR "pensions"[tw]) AND ("claimant"[tw] OR "claimants"[tw] OR "claim"[tw] OR "claims"[tw])

7. "work disablement"[tw]

8. "work disability"[tw]

9. "working disability"[tw]

10. "work absence"[tw]

11. **(**"injured"[tw] OR "injury"[tw] OR "accident"[tw] OR "sickness"[tw] OR "sick"[tw] OR "disability"[tw] OR "disabled"[tw] OR "disablement"[tw] OR "invalid"[tw] OR "invalidity"[tw] OR "absent"[tw] OR "absence"[tw]**) AND (**"worker"[tw] OR "workers"[tw] OR "employee"[tw] OR "employees"[tw]**)**

12. **(**"sickness"[tw] OR "disability"[tw] OR "disabled"[tw] OR "disablement"[tw] OR "invalid"[tw] OR "invalidity"[tw] OR "absent"[tw] OR "absence"[tw] OR "out of work"[tw] OR "social security"[tw] OR "social insurance"[tw]**) AND (**"benefit"[tw] OR "benefits"[tw] OR "beneficiaries"[tw] OR "beneficiary"[tw] OR "claimant"[tw] OR "claimants"[tw] OR "claim"[tw] OR "claims"[tw] OR "pension"[tw] OR "pensions"[tw] OR "pensioners"[tw] OR "pensioner"[tw] OR "compensation"[tw] OR "compensations"[tw]**)**

13. "Insurance, disability"[tw]

14. "disability insurance"[tw]

15. **(**"workman's"[tw] OR "workman"[tw] OR "workmans"[tw] OR "workmen's"[tw] OR "workmen"[tw] OR "workmens"[tw] OR "worker's"[tw] OR "worker"[tw] OR "workers"[tw]**) AND (**"compensation"[tw] OR "compensations"[tw]**)**

16. "case management"[tw]

17. "case manager"[tw]

18. "absence management"[tw]

19. "assertive community treatment"[tw]

20. "management programme"[tw]

21. "management program"[tw]

22. **(**"project"[tw] OR "programme"[tw] OR "program"[tw] OR "model"[tw] OR "interview"[tw] OR "interviews"[tw] OR "plan"[tw] OR "measure"[tw] OR "measures"[tw] OR "intervention"[tw] OR "interventions"[tw] OR "rehabilitation"[tw] OR "management"[tw]**) AND (**"co ordinator"[tw] OR "coordinator"[tw] OR "coordination"[tw] OR "co ordination"[tw] OR "coordinated"[tw] OR "co ordinated"[tw] OR "multidisciplinary"[tw] OR "multi disciplinary"[tw] OR "multi professional"[tw] OR "multiprofessional"[tw] OR "multimodal"[tw] OR "multi modal"[tw] OR "integrated"[tw] OR "patient centred"[tw] OR "patient focused"[tw] OR "individual"[tw] OR "individually"[tw] OR "face to face"[tw] OR "one on one"[tw] OR "in person"[tw] OR "personal"[tw] OR "personally"[tw] OR "customized"[tw] OR "customised"[tw] OR "tailored"[tw] OR "on the job"[tw] OR "work focused"[tw] OR "work related"[tw] OR "workplace"[tw] OR "work location"[tw] OR "work-site"[tw] OR "work place"[tw] OR "job site"[tw] OR "worksite"[tw] OR "occupational"[tw] OR "vocational"[tw] OR "return to work"[tw] OR "counselling"[tw] OR "reintegration"[tw]**)**

22. "rehabilitation"[tw] **AND (**"project"[tw] OR "programme"[tw] OR "program"[tw] OR "plan"[tw] OR "model"[tw] OR "measure"[tw] OR "measures"[tw] OR "intervention"[tw] OR "interview"[tw] OR "interviews"[tw] OR "intervention"[tw] OR "interventions"[tw] OR "management"[tw]**)**

23. "randomized controlled trial"[pt]

24. "controlled clinical trial"[pt]

25. "randomized"[tiab]

26. "randomised"[tiab]

27. "randomly"[tiab]

28. "trial"[ti]

29. "clinical trials as topic"[mesh: noexp]

30. OR / 1- 15

31. OR / 16 - 22

32. OR / 23 - 29

33. AND / 30 – 32
